# Inducing mineral precipitation in groundwater by addition of phosphate

**DOI:** 10.1186/1467-4866-12-8

**Published:** 2011-10-26

**Authors:** Karen E Wright, Thomas Hartmann, Yoshiko Fujita

**Affiliations:** 1 Idaho National Laboratory, PO Box 1625, MS 6188, Idaho Falls, ID 83415-6188, USA; 2 University of Nevada, Las Vegas, 4505 S. Maryland Parkway, Box 454009, Las Vegas, NV 89154-4009, USA; 3 Idaho National Laboratory, PO Box 1625, MS 2203, Idaho Falls, ID 83415-2203, USA

## Abstract

**Background:**

Induced precipitation of phosphate minerals to scavenge trace elements from groundwater is a potential remediation approach for contaminated aquifers. The success of engineered precipitation schemes depends on the particular phases generated, their rates of formation, and their long term stability. The purpose of this study was to examine the precipitation of calcium phosphate minerals under conditions representative of a natural groundwater. Because microorganisms are present in groundwater, and because some proposed schemes for phosphate mineral precipitation rely on stimulation of native microbial populations, we also tested the effect of bacterial cells (initial densities of 10^5 ^and 10^7 ^mL^-1^) added to the precipitation medium. In addition, we tested the effect of a trace mixture of propionic, isovaleric, formic and butyric acids (total concentration 0.035 mM).

**Results:**

The general progression of mineral precipitation was similar under all of the study conditions, with initial formation of amorphous calcium phosphate, and transformation to poorly crystalline hydroxylapatite (HAP) within one week. The presence of the bacterial cells appeared to delay precipitation, although by the end of the experiments the overall extent of precipitation was similar for all treatments. The stoichiometry of the final precipitates as well as Rietveld structure refinement using x-ray diffraction data indicated that the presence of organic acids and bacterial cells resulted in an increasing *a *and decreasing *c *lattice parameter, with the higher concentration of cells resulting in the greatest distortion. Uptake of Sr into the solids was decreased in the treatments with cells and organic acids, compared to the control.

**Conclusions:**

Our results suggest that the minerals formed initially during an engineered precipitation application for trace element sequestration may not be the ones that control long-term immobilization of the contaminants. In addition, the presence of bacterial cells appears to be associated with delayed HAP precipitation, changes in the lattice parameters, and reduced incorporation of trace elements as compared to cell-free systems. Schemes to remediate groundwater contaminated with trace metals that are based on enhanced phosphate mineral precipitation may need to account for these phenomena, particularly if the remediation approach relies on enhancement of *in situ *microbial populations.

## Introduction

The promotion of phosphate mineral precipitation in order to sequester inorganic contaminants has gained interest in recent years (e.g., [1-5]). Immobilization of contaminants *in situ *is an attractive option at many sites because the vast quantities of affected media, often with low pollutant concentrations, make excavation or extraction of the contamination infeasible. Phosphate minerals are advantageous for use in sequestration because they are poorly soluble in many environments and they can incorporate a wide variety of elements. In some cases the sequestration may occur by precipitation of the contaminant in primary minerals (e.g. U in autunite [Ca(UO_2_)_2_(PO_4_)_2_•10-12(H_2_O)], or Pb in pyromorphite [Pb_5_(PO_4_)_3_Cl]). However, because metal or radionuclide contaminants are often at trace levels, coprecipitation with calcium phosphate minerals is common, as calcium is frequently the dominant cation available to react with phosphate in the subsurface. Hydroxylapatite [Ca_5_(PO_4_)_3_(OH)] (HAP) in particular garners attention because of its well-known ability to immobilize trace metals by coprecipitation or solid solution formation [6] as well as by adsorption [7,8]. Sequestration in HAP is especially appealing for radionuclide contaminants for several reasons. Actinides (tri- and tetravalent), Cs, and Sr can be incorporated into the HAP structure [6,9-11], HAP exhibits rapid defect annealing when subject to radiation damage [10] and HAP can be extremely long-lived, as evidenced by the presence of ~2Ga apatites found near the Oklo natural reactor [12].

Phosphate is frequently a limiting nutrient for biological activity in natural terrestrial subsurface systems [13]. Consequently enhanced phosphate mineral precipitation for contaminant immobilization will require the engineered addition and dissemination of phosphate. This is a significant challenge, as the direct addition of high concentrations of soluble inorganic phosphate can result in rapid precipitation and clogging of injection wells [14]. In light of this, several investigators have proposed alternative methods of introducing and distributing phosphate in the subsurface. The use of long-chain polyphosphate to promote uranium immobilization is one option that has been considered [14], and another is the application of the compound glycerol-3-phosphate for degradation by native subsurface microorganisms [15,16]; both cases are examples of "slow release" or *in situ *generation of orthophosphate by degradation of precursor compounds. By moving the location of the introduction of the phosphate beyond the injection well, a wider zone of treatment may be realized and injection well clogging mitigated. An analogous approach has been proposed for immobilization of trace contaminants within carbonate minerals, where bicarbonate ion is generated *in situ *by hydrolysis of urea [17].

Regardless of the mechanism by which reactive phosphate is introduced into the subsurface, the effectiveness of such precipitation-based schemes for remediation of trace contaminants will depend on the specific mineral precipitates generated and their rates of formation. In studies of calcium phosphate mineral precipitation in constant composition experiments conducted over a pH range of 6-9, Zawacki et al. reported that the products were non-stoichiometric apatites, with Ca/P ratios ranging from 1.49 to 1.65 [18]. Precipitation of HAP occurring at high supersaturation has been shown to proceed by steps from the initial precipitation of amorphous calcium phosphate (ACP; Ca_3_(PO_4_)_1.87_(HPO_4_)_0.2_) to final crystallization of HAP [19-21]. Feenstra and de Bruyn (1979) later found that ACP proceeds to HAP via the intermediate formation of octacalcium phosphate (OCP; Ca_8_H_2_(PO_4_)_6_·5H_2_O), which heterogeneously nucleates onto ACP [22]. In experiments at near-neutral pH and 25°C with a Ca/P molar ratio of 1.67 and a saturation index (relative to HAP) of 20.9, Borkiewicz et al. (2010) found that an initial precipitate consisting primarily of ACP with some brushite transformed over the course of seven days to primarily brushite with small amounts of ACP and poorly crystalline HAP. However, at lower levels of supersaturation (10^5 ^- 10^9^, relative to hydroxylapatite), some researchers report that there are no such precursor phases [23], while others state that even at low degrees of supersaturation, HAP never forms homogeneously [24].

In natural systems, organic solutes may also be present and impact the precipitation of HAP. In particular, organic molecules with numerous carboxylate groups, such as humic and fulvic acids, have been shown to significantly reduce precipitation rates even at low concentrations, e.g. 0.25 - 5 ppm [25]. In heterogeneous nucleation systems, it is thought that such molecules adsorb onto seeds and block sites for growth [26]. Organic acids also impact HAP precipitation under non-seeded conditions (homogeneous precipitation). Investigations comparing the effects of 1 mM concentrations of citrate and acetate showed that the presence of citrate resulted in decreased crystal size, higher content of impurities, and greater incorporation of carboxylate groups into the crystal structure, relative to acetate [27]. In addition, the degree of supersaturation required to induce precipitation was higher in solutions containing 1 mM citrate (saturation index = 11.73) than in solutions containing 1 mM acetate; the latter were not significantly different from the organic acid free controls (saturation index = 10.93) [28]. In experiments with 8 mM oxalate, HAP formation was almost completely inhibited due to the precipitation of Ca-oxalate [29].

Phosphate mineral precipitation and dissolution has also been reported to be influenced by the presence of microbial cells. Ca-phosphates and struvite (NH_4_MgPO_4_· 6H_2_O) have been reported to precipitate from culture medium and grow on the outer membrane of Gram-negative bacteria [30]. In experiments on precipitation of Ca-phosphates on agar plates in the presence of *Ramlibacter tataouinensis*, poorly crystallized minerals with low Ca/P ratios formed in the periplasm while nanocrystalline HAP formed inside the cell [31]. Hutchens et al. (2006) reported that when HAP was introduced into systems with both indirect and direct contact with *Bacillus megaterium*, dissolution rates increased, suggesting that the cells decreased the mineral stability, although the rate increase was less in the presence of direct contact between the cells and the HAP [32]. In experiments designed to investigate the impacts of non-metabolizing bacterial cells (gram-positive and gram-negative) on precipitation of phosphate minerals, Dunham-Cheatham et al. (2011) determined that although U-phosphate minerals exhibited heterogeneous nucleation at higher saturation states, Ca-phosphate minerals showed no evidence of heterogeneous nucleation, irrespective of the saturation states tested [33]. However, they noted that at the highest saturation states tested (saturation indices with respect to HAP of 8.29 and 8.34), Ca removal from solution was lower in biotic systems than in abiotic controls, an observation they attributed to the complexation of Ca by bacterial exudates.

The majority of the studies reported in the literature and cited above were conducted in "clean" systems where the precipitation medium was limited almost exclusively to the reactants (calcium, phosphate, counter ions, and selected solutes for testing-- e.g., organic acids), or within bacterial culture media. The objective of the present study was to examine the precipitation of calcium phosphate minerals under conditions more representative of an actual groundwater site. We conducted experiments using a synthetic groundwater (SGW) simulating the composition of the Eastern Snake River Plain Aquifer (ESRPA) in Idaho. This SGW has been used as the medium for another study [34] of the microbial degradation of triethylphosphate (TEP) by mixed cultures derived from the subsurface at the Idaho National Laboratory (INL), a U.S. Department of Energy facility located above the ESRPA. TEP was under evaluation as a biodegradable precursor compound (e.g., an alternative to glycerol-3-phosphate) for slow release of phosphate, as a means for immobilizing contaminants such as ^90^Sr^2+ ^in phosphate minerals.

In our studies with TEP, we observed that despite accumulation of dissolved phosphate in the SGW at concentrations far in excess of what would be expected based on equilibrium considerations, detectable mineral precipitation did not occur [34]. To support interpretation of those results, we decided to determine the threshold concentration of soluble phosphate necessary in order to induce mineral precipitation in the SGW, and then conducted experiments to examine whether the addition of a trace organic acid mixture or bacterial cells affected the course of mineral precipitation and the identity of the solid products. The particular trace organic acids had been identified in the TEP degradation experiments; presumably the acids were released by the microbial cultures. We also compared the impacts of having a "low" and "high" cell concentration within the SGW; a scheme relying on microbial degradation to generate phosphate would likely involve enhancement of microbial abundance in the environment. The cells used in these experiments were a strain of *Comamonas testosteroni*; phylogenetic analyses of the mixed culture in the TEP degradation experiments indicated relatives of this gram-negative soil bacterium were present in the enrichment [34].

## Materials and methods

### Chemicals and Materials

All chemicals used in the experiments were ACS reagent grade. Water was 0.2 μm filtered "nanopure" grade (18 megohms-cm; Barnstead, Dubuque, IA).

### Synthetic Groundwater Composition

Synthetic groundwater (SGW) was formulated to mimic the ESRPA groundwater composition (Table 1). It should be noted that elevated in-situ *p*_CO2 _in the ESRPA causes CO_2 _to exsolve, pH to rise, and calcite to precipitate upon sampling under atmospheric pressure conditions. The SGW was formulated to reproduce in-situ pH conditions (pH 7.5, adjusted using HCl). Therefore, under experimental conditions of atmospheric pressure, the SGW contains less bicarbonate than the real ESRPA groundwater in order to preserve stability with respect to atmospheric *p*_CO2_.

**Table 1:** Initial synthetic groundwater composition

Analyte	mM
Na^+^	2.04

Mg^2+^	0.72

Ca^2+^	1.50

K^+^	0.20

HCO_3 _^-^	0.65*

SO_4_^2-^	0.26

Cl^-^	5.62

NO_3_^-^	0.41

Sr^2+^	0.01

pH	7.5

* Note that in bringing SGW to pH 7.5, CO_2 _is lost to the atmosphere. Actual bicarbonate concentration at pH 7.5 is about 0.21 mM. Indicated chloride concentration includes that contributed by added HCl to achieve desired pH.

### Microbial Culture Preparation

*C. testosteroni *(ATCC 11996) was grown overnight in Nutrient Broth with 2% yeast extract. The mid-to-late log phase culture was harvested and washed three times by centrifugation (14,000 × *g*; 2 minutes) at 4°C and was re-suspended in sterile distilled water. The final cell concentration in the suspension was adjusted as needed to achieve the desired cell density in the experiments by the addition of 1 mL of cell suspension.

### Precipitation Experiments

The impacts of the presence of bacterial cells (*C. testosteroni*) and of a trace mixture of organic acids on the course and products of phosphate mineral precipitation in the SGW were investigated in batch reactor systems. The selection of particular organic acids and their concentrations was based on observations from the TEP biodegradation experiments [34]. Because the solid phosphate phases that form initially are not necessarily the phases that persist over time [21,35], the intention was to evaluate the course of precipitation over one week at 25°C. To determine an appropriate amount of initial phosphate such that the onset of precipitation was detectable within a 24 - 72 hr window, a preliminary experiment was conducted with varying amounts of NaH_2_PO_4 _added to vessels containing SGW. Based on the results of this experiment, and using a > 3% drop in aqueous phosphate concentration as a target indicator of precipitation, an initial phosphate concentration of approximately 1.6 mM was selected for the time course experiments. The different treatments evaluated are shown in Table 2.

**Table 2:** Treatments for Precipitation Experiments

Treatment	Description
Abiotic Control Treatment (AC)	SGW only

Organic Acid Treatment (OA)	SGW with organic acid mixture*

"Low" Concentration Cell Treatment (LC)	SGW with 10^5 ^cells mL^-1^

"High" Concentration Cell Treatment (HC)	SGW with 10^7 ^cells mL^-1^

*The organic acid composition in Treatment OA was as follows: propionate (0.012 mM), isovalerate (0.01 mM), formate (0.003 mM), butyrate (0.01 mM). Propionate, formate and butyrate were added to the SGW as sodium salts. Isovalerate was added as an ammonium salt.

Possible calcium phosphate precipitation products and their calculated saturation indices for the initial conditions are indicated in Table 3 where Q is the ion activity product and K_so _is the mineral's equilibrium solubility product. These thermodynamic calculations were made using vMinteq v. 2.61 [36,37] with the thermo.vdb thermodynamic database, the comp_2008 component database, and the type 6 solids database [38]. Amorphous calcium phosphates AM1 and AM2 (both phases are Ca_3_(PO_4_)_1.87_(HPO_4_)_0.2_) are described in Christoffersen et al. (1989, 1990) [39,40]. Note that β tricalcium phosphate cannot precipitate from low temperature aqueous solutions [41]; however, when some substitution of Ca by Mg is permitted, this phase can precipitate as whitlockite (Ca_18_Mg_2_H_2_(PO_4_)_14_.

**Table 3:** Possible calcium phosphate phases and their calculated saturation indices (log Q/K_so_)

		log Q/K			
**AM1^1^** **Ca_3_(PO_4_)_1.87_(HPO_4_)_0.2_**	**AM2^1^** **Ca_3_(PO_4_)_1.87_(HPO_4_)_0.2_**	**Ca_3_(PO_4_)_2 _β^2^**	**OCP^1^** **Ca_8_H_2_(PO_4_)_6 _**·**5H_2_O**	**Brushite^2^** **CaHPO_4_**·2H_2_O	**HAP^2^** **Ca_5_(PO_4_)_3_(OH)**

-0.126	2.624	3.294	3.568	0.239	11.838

^1 ^Thermodynamic data used in Minteq log Q/K_so _calculations comes from Christofferson et al., 1990

^2 ^Thermodynamic data used in Minteq log Q/K_so _calculations comes from Smith et al., 2003

Triplicate batch systems were set up for each treatment and each of three time points (60 minutes, 24 hrs, and 7 days), for a total of 9 bottles per treatment, to allow sacrifice and collection of all of the newly precipitated solids for analysis at the designated times. For each replicate, 100 mL of SGW (pH 7.5) was added to an acid-washed and autoclaved 250 mL polymethyl pentene bottle. Organic acids were added to the OA bottles, and suspensions of washed *C. testosteroni *cells were added to the LC and HC treatments with targeted initial average cell densities of 10^5 ^and 10^7 ^cells mL^-1^, respectively. Then 1.6 mL of 0.1 M NaH_2_PO_4 _(pH 7.5) was added to each bottle. The closed bottles were incubated on a shaker table in a temperature-controlled (25°C) chamber.

Each bottle was sampled immediately after preparation to determine the elemental composition of the aqueous phase. In addition, aqueous samples (1.5 mL) were collected at 60 minutes, 24 hours, and daily until seven days had elapsed. Each sample was centrifuged for 10 minutes at 12,700 × *g*. The supernatant was filtered (0.2 μm) prior to acidification (nitric acid) and analysis of elemental composition. The pH was measured periodically throughout the experiment.

Samples for cell counts (1 mL for HC treatments, 10 mL for LC treatments) by fluorescent microscopy were also removed at the beginning and end of the experiment, and immediately preserved with 2% formaldehyde and refrigerated until processing. All sample transfers were conducted using sterilized implements and containers.

For solid (precipitated minerals and/or cells) collection, the supernatant was removed after centrifuging (8,000 × *g*, 20 minutes) and then the solids were washed 5 times with ethanol and repeated centrifuging to remove residual salts. The solids collected from the three replicates for a given treatment and time period (e.g. T1, 24 hours) were combined to attain sufficient mass for analysis.

### General Analytical Techniques

#### ICP-AES

Analysis of aqueous samples for Na, K, Ca, Mg, Sr, P, and S was carried out by ICP-AES (iCAP 6000, Thermo Fisher Scientific, Waltham, MA).

#### Scanning Electron Microscopy

Solids (in an ethanol slurry) were mounted onto silicon plates, dried, and coated with a thin layer of platinum. Scanning electron microscopy (SEM) of the solids was performed using an FEI Q650 with a tungsten field emission gun in environmental mode using a large field detector. Accelerating voltage was 5 KeV.

#### X-Ray Diffraction

The ethanol-washed solids were mounted on oriented silicon plates and allowed to air dry before analysis by X-ray diffraction (XRD) using a Bruker D8 Advance X-ray diffractometer (Bruker AXS, Congleton, Cheshire, UK) with Cu Kα_1/2 _emission using a Goebel mirror. The accelerating voltage was 40 KV with a current of 30 mA and the step size was 0.02 degrees with an integration time of 2 seconds.

#### Rietveld Structure Refinement

Rietveld structure refinement was performed on diffraction patterns obtained from solids collected at the end of the experiment (7 days) to confirm phases, refine lattice parameters and estimate crystallite dimensions. Topas 4.2 (Bruker AXS) was used for peak profile fitting and application of analytical Voigt functions to fit the diffracted peak profiles.

#### Microbial Enumeration

Microbial cells in the formaldehyde-preserved samples were enumerated using direct microscopic counts. Cells were stained with 0.01% acridine orange, filtered onto black 25 mm 0.2 μm polycarbonate filters, and counted by epifluorescent microscopy using standard protocols [42].

## Results

### Changes in Solution Chemistry

**Figure 1:**
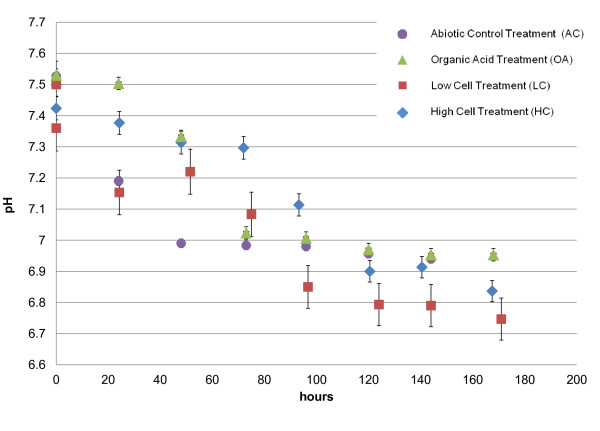
**Average pH over the course of the experiment**. Error bars show two standard deviations based on triplicate analysis.

The pH decreased in all of the treatments over the 7 days of observation (Figure 1), as would be expected with HAP precipitation. However, the rate of pH change varied for the treatments. In the abiotic control (AC), most of the pH change occurred within the first 48 hrs and then the pH stabilized to just below 7 by 120 hrs. In contrast, in the organic acid (OA) treatment the pH dropped more slowly, although it mirrored the behavior of the AC treatment after 72 hours.

The low cell (LC) treatment showed a continually dropping pH for the first 96 hours and the final pH was lower than in the AC or OA treatments. In comparison the high cell (HC) treatment showed a delayed response, but it too reached a pH value lower than the AC and OA treatments and was indistinguishable from the LC treatment by the end of the 7 days. The lower pH in the cell-containing treatments could have been due to the release of amino acids or other organic acids from the cells during the experiments; both LC and HC treatments exhibited a pungent amine-like odor at the conclusion of the experimental period. However, high performance liquid chromatography assays for common organic acids (citrate, butyrate, oxalate, propionate, formate, acetate, succinate, and malate) conducted on aliquots from the treatments after both 1 and 7 days did not detect any of these compounds at levels above the method detection limit (0.1 mM except for isobutyrate (0.18 mM) in one of the three replicates for the LC treatment after one day; data not shown).

**Figure 2:**
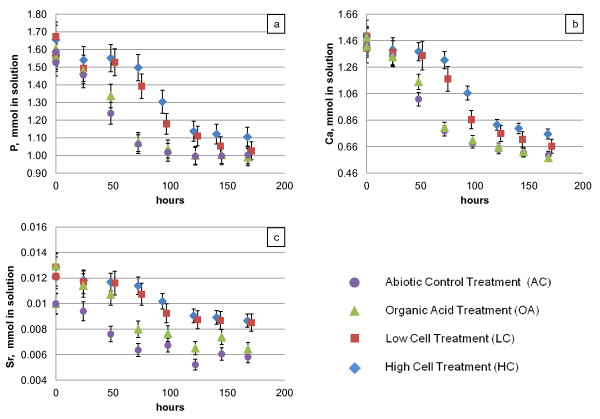
**Changes in ion concentration in solution over the course of the experiment: (a) phosphorus (b) calcium, and (c) strontium**. Error bars show two standard deviations based on triplicate analysis.

With respect to soluble P concentrations (Figure 2a), all of the treatments behaved similarly the first 24 hours, but by 48 hours, there were significant differences between the cell-containing treatments and the cell-free treatments. Aqueous P concentrations for cell treatments (LC and HC) were elevated relative to cell-free treatments (AC and OA), particularly for the first 72 hours, and they remained higher for much of the duration of the experiment, until the last 2 days, when they appeared to have "caught up to" the cell-free treatments. There was no statistically significant difference in P concentrations between the two cell-containing treatments nor between the two cell-free treatments.

The trends in Ca and Sr concentrations (Figures 2b and 2c) were similar to the trend observed for P--that is, removal of all 3 elements from solution is delayed in the cell-containing treatments as compared to treatments without cells. Similarly to the situation for P, there does not appear to be a statistical difference between the two cell treatments nor between the cell-free treatments with respect to Ca removal. However there are differences between treatments with respect to Sr removal. Until the final time point, the AC treatment removes more Sr from solution than any other treatment (Figure 2c). The difference between treatments becomes more obvious if we consider the Ca/Sr ratio in solution. By the end of the experiment, the ratio of Ca/Sr remaining in solution was highest in the AC treatment (molar ratio [Ca]/[Sr] = 102); in comparison the OA, LC, and HC treatments had final solution [Ca]/[Sr] values of 89, 78, and 87, respectively. In other words, the OA, LC and HC treatments apparently resulted in less Sr uptake into the solids compared to the abiotic control. The decreases in aqueous Mg concentrations measured were very small (~-0.05 mM, equivalent to 7%) and uniform for all treatments (data not shown). Within the resolution of our methods, concentrations of Na, K, and S for all treatments were unchanged during course of the experiment.

### Characterization of Solids

No solids were observed to have formed after 60 minutes in any of the treatments. After 24 hours, precipitated solids were visible and could be collected from the AC treatment. Solids were also present in the OA and LC treatments, but in both cases the collected solids re-dissolved in the ethanol used for washing, and could not be subsequently characterized by XRD or SEM. Precipitates could not be collected from the HC treatment until after 72 hours.

**Figure 3:**
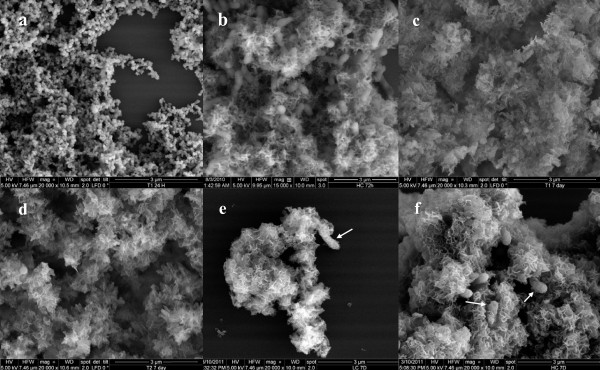
**Representative SEM images of solids collected from the four treatments during the course of the experiment: (a) treatment AC after 24 hours, (b) treatment HC after 72 hours, (c) treatment AC after 7 days, (d) treatment OA after 7 days, (e) treatment LC after 7 days, and (f) treatment HC after 7 days**. Arrows in (e) and (f) indicate where precipitate appears to occur on bacterial cells. Scale bar for all images is 3 micrometers.

SEM examination of the AC solids collected after 1 day showed agglomerations of roughly spherical particles on the order of 100 nm in diameter (Figure 3a). The XRD pattern of the solids was consistent with that reported by Li and Weng [43] for amorphous calcium phosphate (ACP). The HC solids at 72 hours also showed a similar XRD pattern (data not shown), although the SEM imaging showed that the precipitates were more needle-like (Figure 3b). Bacterial cells were enmeshed within the precipitated material.

**Figure 4:**
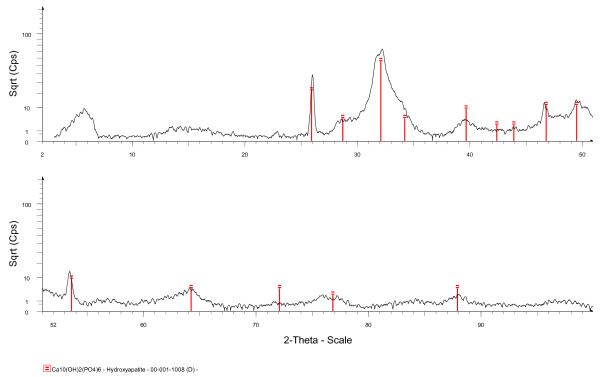
**X-ray diffraction pattern of precipitates from the AC treatment collected after 7 days**.

After 7 days, solids were collected from all four treatments. Imaging of the 7-day solids from all of the treatments by SEM showed a morphology like crinkled paper (Figures 3c through3f). Bacterial cells were visible amongst the precipitates from the LC and HC treatments, and precipitates appeared to coat some of the bacterial surfaces (arrows in Figures 3e and 3f). Figure 4 shows an X-ray diffraction pattern for precipitates collected from the AC treatment at the conclusion of the experiment. The pattern is consistent with poorly crystalline HAP, with no significant precipitation of any other phase. Elevated counts seen at ~6^o ^2θ are caused by the sample holder. The pattern collected from the specimens representing the other three treatments looked similar and are not shown here.

Results from Rietveld analysis performed on diffraction patterns from 7-day solids are shown in Table 4. The results confirmed the poorly crystalline nature of the samples; the lack of adequate crystallinity precluded determination of the crystal size. When compared with natural hydroxylapatite, which has lattice parameters of *a *= 9.417 Å and *c *= 6.875 Å, respectively [44], the results clearly showed that lattice parameter *a *became substantially larger with the following progression AC < OA < LC < HC while lattice parameter *c *became somewhat smaller (AC≈OA > LC = HC). The overall effect is an increasing cell volume in the sequence AC < OA < LC < HC. The 7-day samples were analyzed a second time by XRD 10 months after the initial analysis. There were no significant changes in the X-ray diffraction patterns generated by the AC, OA, or LC treatments; however, although the samples had been stored dry, the second analysis of the HC treatment produced a diffraction pattern completely devoid of peaks, suggestive of a phase lacking any far- or medium-range ordering.

**Table 4:** Cell dimensions of the HAP precipitates at day 7

Sample	Refinement Residual Rwp %	Lattice Parameter *a *(Å)	Lattice Parameter *c *(Å)	Cell Volume (Å^3^)
Abiotic Control Treatment (AC)	12	9.4563 ± 0.0013	6.8617 ± 0.0011	531.38 ± 0.17
Organic Acid Treatment (OA)	10.8	9.4757 ± 0.0018	6.8680 ± 0.0015	534.05 ± 0.24
Low Cell Treatment (LC)	10.2	9.4976 ± 0.0017	6.8582 ± 0.0013	535.75 ± 0.21
High Cell Treatment (HC)	8.8	9.5229 ± 0.0031	6.8565 ± 0.0023	538.48 ± 0.39

For pure systems, the degree to which HAP deviates from stoichiometry can be described by the Ca/P molar ratio. Perfectly stoichiometric HAP has a Ca/P molar ratio of 1.67. Because our precipitated phase contains Mg and Sr in addition to Ca, it is reasonable to examine the divalent cation to phosphate ratio (i.e., (Ca + Mg + Sr)/P). The assumption that the major ions accounting for the phase's stoichiometry include Ca, Mg, Sr, and P is reasonable because although carbonate can substitute into the crystal structure, it would result in a smaller *a *lattice parameter [45], not the larger *a *parameter as we observe with our solids. This suggests that if carbonate is present in the crystal structure, the effect is dominated by substitution of HPO_4_^2- ^(see Discussion). Although some substitution of chlorine for hydroxyl is possible in these solids, it would not change the structure type or the overall phase identification.

**Figure 5:**
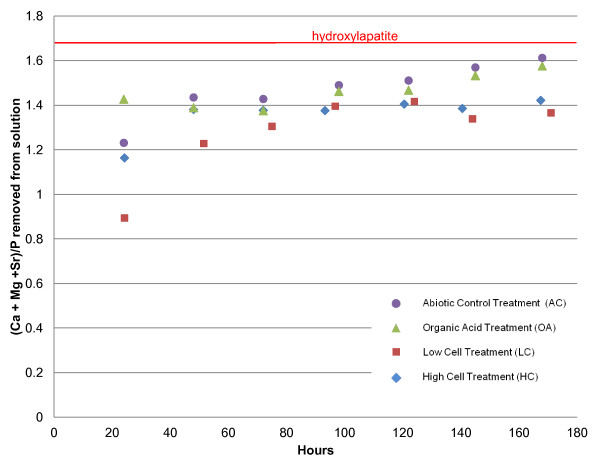
**The evolution of the divalent cation/P ratio of ions removed from solution for all treatments**.

The trends in the divalent cation/P ratios for each of the four treatments are presented in Figure 5, which shows that stoichiometric HAP is most closely approached by the OA and AC treatments. Even in the last 50 hours of the experiments their divalent cation/P ratios still appeared to be increasing toward 1.67 (at hour 168, the ratio for the OA treatment is 1.58, while the AC treatment's is 1.61), while the ratio for the treatments containing cells appeared fixed around 1.4, where it had been for the last three days of the experiment.

### Cell Growth

Although the cells used for inoculation in the LC and HC treatments had been washed with deionized water and were not provided with growth substrates, cell populations nonetheless appeared to increase over the course of the week long experiment for the LC treatment. The electron and carbon donor(s) necessary to support the observed growth are unknown. As noted previously, analyses for organic acids at day 1 and day 7 did not indicate accumulation of such compounds although it is possible that they were released by cell lysis at concentrations below the method detection limit and/or that they were released but rapidly consumed. During the cell enumeration procedure, it was noted that the cells at day 7 appeared smaller than at day zero. Cell counts rose from 4.28 ± 0.87 × 10^5 ^at time zero to 1.11 ± 0.21 × 10^6 ^mL^-1 ^at 7 days. In the HC treatments, the counted numbers of suspended cells decreased from 2.31 ± 0.38 × 10^7 ^to 3.76 ± 0.27 × 10^6 ^mL^-1^, but these values likely do not reflect the true biomass as an extensive biofilm was observed to develop on the bottom of the HC reactor vessels during the week-long experiment. The increased cell density in the LC treatment could explain why the two cell-containing treatments did not appear to be significantly different from each other with respect to precipitation progress (Figure 2) or final product composition (Figure 5).

## Discussion

Calcium phosphate precipitation in the synthetic groundwater in the absence of organic acids or cells appeared to proceed largely as expected based on the reports of other studies conducted under similar conditions (e.g., level of saturation, pH) but in aqueous media of simpler composition. The mineral phase that precipitated after 1 day (amorphous calcium phosphate) was not the same as the one identified at the end of 7 days (poorly crystalline HAP). Although we were not able to detect them with our experimental design, it is possible that other transient phases besides ACP were formed prior to the crystallization of HAP. In experiments conducted at ambient temperature and near-neutral pH in a medium of 100 mM calcium acetate and 60 mM ammonium phosphate (conditions resulting in a saturation index relative to HAP of 20.9 and a Ca/P molar ratio of 1.67, compared to our values of 11.8 and 0.94, respectively), Borkiewicz et al. (2010) detected the formation of only brushite and ACP during an 8 hr observation period, using *in situ *monitoring with synchrotron XRD [21]. However, in a similar seven-day precipitation experiment with *ex situ *monitoring by XRD, Borkiewicz et al. (2010) observed initial formation of a precipitate mixture composed predominantly of ACP, with a small amount of brushite. By the experiment's conclusion, the amorphous precipitate had largely transformed into brushite with small amounts of poorly crystalline HAP and some ACP remaining.

The addition of the mixture of organic acids did not appear to impact precipitation progress relative to the abiotic control; the solute concentration profiles were almost identical (Figure 2), as was the composition of the final mineral phase as estimated from solution chemistry (Figure 5). The pH decrease initially lagged, but by 72 hours the trend for the OA treatment mirrored the profile for the abiotic control. The physical appearance (Figure 3d) of the final solid was also indistinguishable from that of the abiotic control. However, the OA cell volume was somewhat larger than the cell volume of the AC precipitate (Table 4)--a finding that is contrary to what van der Houwen et al. (2003) reported based on studies using a higher concentration of organic acids (1 mM) in solution. The organic acid mixture of propionate, isovalerate, formate and butyrate was added at a total concentration of 0.035 mM; this was equivalent to 0.13 mM dissolved organic carbon (DOC) or 1.5 mg-C L^-1^. This level of DOC is higher than the concentrations reported by Amjad and Reddy (1998) [25] to slow the rate of HAP precipitation, but Amjad and Reddy were studying the effects of humic compounds, rather than simple monocarboxylic acids like those included in this study. Our findings support the reports of van der Houwen et al. (2003), that the number of carboxylate groups is more important than the bulk DOC concentration with respect to inhibition of HAP precipitation.

The addition of cells to the precipitation medium appeared to have a more dramatic impact than the organic acids. During the first 24 hours, the extent of precipitation in the cell-containing treatments appeared to be indistinguishable from the cell-free treatments, as indicated by the P and Ca profiles (Figures 2a and 2b) respectively. However, after the first day the rate of precipitation in the cell-containing treatments apparently slowed, and removal of Ca, Sr and P from solution was less in the LC and HC treatments compared to the AC and OA treatment, until near the end of the experiment. The reason for the temporary lag in precipitation is unknown. One possibility is the production of extracellular polymeric substances (EPS) by the microbial cells. EPS can inhibit mineral precipitation by reducing diffusion rates and creating microgradients of solutes at the mineral surface, and they can also bind divalent metals, such as Ca^2+ ^and Mg ^2+^, thus decreasing effective saturation in the bulk phase [46]. The resulting constraint on mass transfer of the reactants to the mineral surface would result in slowed precipitation rates. As noted previously, for their experiments with initial saturation states closest to our conditions, Dunham-Cheatham et al. (2011) reported decreased aqueous Ca removal in the presence of cells compared to abiotic controls and attributed this to formation of Ca complexes with bacterial exudates. However, their experiments were conducted primarily over the span of 2-3 hours; the longest experiments performed were 48 hours [33]. In our experiments, between 2 and 5 days we too observed significant differences in Ca removal between the cell-containing and cell-free experiments (Figure 2b). However, the two conditions converged by the end of our 7 day experiments. EPS production would be consistent with the observed development of a biofilm in the HC treatment reactors. In the LC reactors, no biofilm formation was visible, but this may have been associated with the lower initial cell density.

With respect to observed concentrations of phosphate in solution, in experiments such as ours there is the potential for internal phosphate reserves within the cells to play a role. Dunham-Cheatham et al. (2011) noted some contribution of phosphate from cells in their experiments. However, in our studies the concentration of phosphate added was relatively high (~1.6 mM) and the cell density was relatively low (maximum 2.31 × 10^7 ^cells/mL). Assuming 6.7fg P per stationary phase cell (based on an estimate for *E. coli*; [47]), the potential P contribution from cells was 300 times smaller than the amount of P added externally as NaH_2_PO_4_, suggesting that the potential phosphorus contribution from the cells was negligible.

One of the most significant differences between the treatments lies in the degree of departure from HAP stoichiometry for the various treatments. As seen in Figure 5, HAP stoichiometry is most closely approached with the AC treatment (divalent cation/P ratio = 1.61), and its greatest departure is with the treatments containing cells (divalent cation/P ratio ~1.4), with the OA treatment having a Ca/P ratio of 1.58. So-called Ca-deficient and other nonstoichiometric apatites have been synthesized in the laboratory, and it has been reported that the lower the HAP stoichiometry, the larger the *a *lattice parameter [45]. The size of the *c *lattice parameter was related more to the pH of formation, with a smaller *c *lattice parameter forming at a pH less than 7 [45]. This is consistent with our data, which shows that the less stoichiometric precipitates are accompanied by the larger lattice parameter *a *(Figure 5, Table 4). The smaller *c *lattice parameter for the cell-containing treatments is likely the result of the lower solution pH measured for those treatments (Figure 1). The expansion of the *a *lattice parameter in non-stoichiometric apatites is attributed to HPO_4_^2- ^and water in the structure (Elliot, 1994). This results in a cation deficiency (lower Ca/P ratio), which is reflected most obviously in the relatively lower amount of calcium incorporated into the HC and LC solids, but this phenomenon would also reduce Sr incorporation into those same solids [48].

The increased departure from stoichiometry for the HAP produced in the cell-containing treatments as compared to the AC and OA treatments may have also been related to the observed loss of crystal integrity for the HC treatment by the time of the second XRD analysis. In natural HAP, the bond between the phosphorus and each oxygen is about 60% covalent, with the remainder consisting primarily of ionic bonding [49]; [50]. However, with non-stoichiometric HAP, calcium ions are removed from the crystal structure and are replaced by ions that are hydrogen-bonded to adjacent ions [49]. In the case of the HC treatment precipitates, it appears that the non-stoichiometry was to such a degree that 10 months after precipitation the X-ray diffraction pattern no longer showed any evidence of medium- or far-range order, although the SEM images looked similar at both timepoints.

## Conclusions

The induced precipitation of hydroxylapatite mineral phases in "clean" laboratory systems (i.e., containing just Ca, phosphate, sodium and chloride) has been observed to require the generation of very high levels of supersaturation (> 10 orders magnitude with respect to HAP) [28] and our results suggest that this will be true for groundwater systems as well. Our results also are consistent with the reports of others that HAP precipitation occurs following the transformation of a precursor phase of amorphous calcium phosphate.

The crystallization of HAP in our experiments proceeded relatively slowly; at the end of the week-long experiments the precipitates in the cell-free treatments still appeared to be evolving toward more stoichiometric, and presumably more stable, HAP. This suggests that the minerals formed initially during an engineered precipitation application for trace element sequestration may not be the ones that control long-term immobilization of the contaminants.

The organic acids that were detected in previous experiments designed to observe phosphate mineral precipitation induced by microbial degradation of TEP were unlikely to have been responsible for the absence of detectable mineral precipitation; rather the amount of phosphate released by the microbes was simply insufficient to achieve the required level of supersaturation. In a natural system where sufficient phosphate can be added however, organic acids may be important, although they are more likely to be humic and fulvic acid type compounds than the simple monocarboxylic acids tested here.

Another finding from our study is that microbial cells at concentrations representative of typical and stimulated groundwaters can impact HAP formation by delaying precipitation, by making the precipitates less stoichiometric, and by reducing the incorporation of trace elements in the HAP as compared to cell-free systems. In addition, such precipitates may exhibit reduced long-term stability. Whether these effects are exerted through intact cells or via soluble organic compounds derived from or released by the cells could not be conclusively determined in our study. Nevertheless, schemes to remediate groundwater contaminated with trace metals that are based on enhanced phosphate mineral precipitation may need to account for such effects, particularly if the remediation approach relies on enhancement of *in situ *microbial populations.

## Competing interests

The authors declare that they have no competing interests.

## Authors' contributions

KW and YF designed and executed the experimental work, and drafted the manuscript. TH conducted Rietveld structure refinement and reviewed the manuscript. All authors reviewed and approved the final manuscript.
